# A nanobody‐horseradish peroxidase fusion protein‐based competitive ELISA for rapid detection of antibodies against porcine circovirus type 2

**DOI:** 10.1186/s12951-021-00778-8

**Published:** 2021-02-01

**Authors:** Yang Mu, Cunyu Jia, Xu Zheng, Haipeng Zhu, Xin Zhang, Haoran Xu, Baoyuan Liu, Qin Zhao, En-Min Zhou

**Affiliations:** 1 Department of Preventive Veterinary Medicine, College of Veterinary Medicine, Northwest A&F University, Yangling, Shaanxi 712100 China; 2 Scientific Observing and Experimental Station of Veterinary Pharmacology and Diagnostic Technology, Ministry of Agriculture, Yangling, Shaanxi 712100 China

**Keywords:** Nanobody-HRP fusion protein, Competitive ELISA, PCV2, Antibody detection

## Abstract

**Background:**

The widespread popularity of porcine circovirus type 2(PCV2) has seriously affected the healthy development of the pig industry and caused huge economic losses worldwide. A rapid and reliable method is required for epidemiological investigation and evaluating the effect of immunization. However, the current methods for PCV2 antibody detection are time-consuming or very expensive and rarely meet the requirements for clinical application. we have constructed the platform for expressing the nanobody(Nb)‑horseradish peroxidase(HRP) fusion protein as an ultrasensitive probe to detect antibodies against the Newcastle disease virus(NDV), previously. In the present work, an Nb-HRP fusion protein-based competitive ELISA(cELISA) for rapid and simple detection antibodies against PCV2 was developed using this platform to detect anti-PCV2 antibodies in clinical porcine serum.

**Results:**

Using phage display technology, 19 anti-PCV2-Cap protein nanobodies were screened from a PCV2-Cap protein immunized *Bactrian camel*. With the platform, the PCV2-Nb15‑HRP fusion protein was then produced and used as a sensitive reagent for developing a cELISA to detect anti‑PCV2 antibodies. The cut‑off value of the cELISA is 20.72 %. Three hundreds and sixty porcine serum samples were tested by both newly developed cELISA and commercial kits. The sensitivity and specificity were 99.68 % and 95.92 %, respectively. The coincidence rate of the two methods was 99.17 %. When detecting 620 clinical porcine serum samples, a good consistent (kappa value = 0.954) was found between the results of the cELISA and those of commercial kits.

**Conclusions:**

In brief, the newly developed cELISA based PCV2-Nb15‑HRP fusion protein is a rapid, low-cost, reliable and useful nanobody-based tool for the serological evaluation of current PCV2 vaccine efficacy and the indirect diagnosis of PCV2 infection.

## Background

In the early 1990s, a naturally occurring specific antibody, discovered in camelids, is known as a heavy chain antibody (HCAb), which lacks the light chain and CH1 regions of the heavy chain [1]. The variable region of HCAb, named as variable domains of the heavy chain of HCAb (VHH), is the smallest antibody unit with a complete antigen-binding function. Its structure is shaped like a rugby ball, with a diameter of about 2.5 nm and a height of about 4 nm, and a molecular weight of approximately only 15 kDa. Therefore, it is also known as “nanobody (Nb)” [2, 3]. The single-domain nature of nanobodies confers many special properties not observed in conventional antibodies, including high affinity, thermal stability, and high yield in microbial production systems [4]. The special structure makes nanobody incline to identify epitopes that are recognized difficultly by traditional antibodies.

PCV2, a non-encapsulated single-stranded circular DNA virus and the smallest animal virus, belongs to *Circoviridae Circovirus*. The virion is 17 to 20 nm in diameter and forms a covalently closed circular structure with icosahedral symmetry and a molecular mass of about 28 kDa [5]. PCV2 is relatively resistant to the environment. It is insensitive to acids and can survive in low pH environments, but it is sensitive to compounds, such as chloroform and other oxidants [6]. PCV2 usually infects 5 to 18 weeks old piglets and is considered to be the main cause of postweaning multisystemic wasting syndrome (PMWS) [7, 8]. In addition to PMWS, PCV2 also causes a series of related diseases that are collectively known as Porcine circovirus-associated disease (PCVAD) [9]. It is considered as an important, economically challenging pathogen on a global scale with comprehensive vaccination schemes in place [10]. The widespread popularity of PCV2 has seriously affected the healthy development of the pig industry and caused huge economic losses worldwide. PCV2 has two main open reading frames, ORF1 and ORF2. ORF1 is predicted to encode a replication-associated protein (Rep) which is essential for the replication of viral DNA, while ORF2 encodes the major capsid protein (Cap) [11]. Cap, the most important immunogenic protein with highly conserved epitopes of PCV2, is responsible for the production of PCV2-specific neutralizing antibodies. It can induce a strong immune reaction against sera from PCV2-positive animals and has been employed for developing vaccines and serodiagnostic assays [12, 13].

Enzyme-linked immunosorbent assay (ELISA) is a simple and rapid technique for the detection and quantification of antibodies or antigens and is widely used in laboratory research, disease diagnosis, and immune level detection. Conventional antibodies, polyclonal and monoclonal antibodies, are often used as reagents in ELISA reactions. In recent decades, different types of ELISAs, including indirect ELISA [14–18], competitive ELISA [19–21], blocking ELISA [22], and double-antigen sandwich ELISA [23, 24], have been widely applied to detect PCV2 antibodies in large-scale blood or feces samples. However, these assays are based on PCV2-specific monoclonal or polyclonal antibodies that require more support cost and exhibit low expression yields and high levels of instability. As a novel antibody, nanobody is simple to prepare, costs less, and can bind to specific epitopes that may not be recognized by conventional antibodies. The fusion of PCV2 specific single domain antibodies (psdAb) and alkaline phosphatase (AP) has been used in indirect ELISA for simple, convenient, and sensitive detection of PCV2 [25]. We have constructed the platform for expressing the nanobody‑HRP fusion protein as an ultrasensitive probe to detect antibodies against the Newcastle disease virus, previously [26]. Here, used the constructed platform and the same approach, a nanobody-HRP fusion protein-based competitive ELISA for rapid and simple detection antibodies against PCV2 was developed to detect anti-PCV2 antibodies in clinical pig serum.

## Materials and methods

### Protein, cells, and vectors

Soluble expressed and purified recombinant PCV2-Cap protein was kindly presented by Dr. Xuehui Cai from Harbin Veterinary Research Institute, Chinese Academy of Agricultural Sciences. HEK 293T cells were cultured in Dulbecco’s Modified Eagle’s Medium (Life Technologies Corp, USA) contained 10 % fetal bovine serum (FBS, Gibco, USA) at 37 °C in 5 % CO_2_. Modified pEGFP-N1 vector(named as pCMV-N1-HRP)with fusion expression of the signal peptide of human Ig kappa chain, HA tag, codon-optimized HRP, and His tag was constructed as previously described [26].

### Experiment animals

Adult *Bactrian camels* were rented from the Minqin Camel farm in Gansu province, China. New Zealand rabbits were purchased from Chengdu Dossy Experimental Animals Co., LTD, China.

### Serum samples

Three hundred and sixty serum samples of PCV2 vaccine immunized pigs or non-immunized pigs, collected from Shiyang Agricultural Group Co. LTD and confirmed with a commercial ELISA kit (Shenzhen Finder Biotech Co., Ltd), were used to develop the cELISA and determine the cut-off value and specificity of the developed cELISA. Positive sera against porcine reproductive and respiratory syndrome virus (PRRSV), porcine pseudorabies virus (PRV), classical swine fever virus (CSFV), transmissible gastroenteritis virus (TGEV), and porcine epidemic diarrhea virus (PEDV) are validated serum samples kept in our laboratory. Six hundred and twenty clinical serum samples from immunized pigs were collected from large-scale farms in Shaanxi, Hebei, Shandong, and Henan provinces.

### Preparation of rabbit anti‐camel IgG antiserum

The *Bactrian camel* blood was collected from the jugular vein and the serum was separated. The serum was diluted by adding equal volume PBS (0.01 mol/L, pH 7.2), then filtered through a 0.45 µm filter membrane and the filtrate was used as a sample to be purified. The sample was slowly added to Protein G Affinity Resin (Genscript, Nanjing, China) purification column and purified according to the instructions. The eluate was collected and neutralized rapidly to pH 7.2–7.4 with Tris-HCl buffer (1 mol/L, pH 8.5). The purified camel serum IgG was analyzed by SDS-PAGE. Adult rabbits were immunized with 1 mg (1 mg/mL) purified camel serum IgG each time and immunized 3 times at a 2-week interval. Freund’s complete adjuvant (Sigma-Aldrich, SA) was used for the first immunization, followed by Freund’s incomplete adjuvant. One week after the last immunization, the blood of the immunized rabbits was collected and the sera were separated. Indirect ELISA (iELISA) was used to detect anti-camel serum IgG antibody titer and unimmunized rabbit serum was used as negative control. In brief, purified camel serum IgG (400 ng/well) was used as coating antigen, and HRP-conjugated goat anti-rabbit IgG (dilution 1:5000, Jackson ImmunoResearch Laboratories, USA) was used as the secondary antibody.

### Bactrian camel immunization and VHH library construction

A 4.5-year-old male *Bactria*n camel was immunized by subcutaneous route with purified recombinant PCV2-Cap protein by 2 weeks interval as described in previous studies [27, 28]. Two milligram Cap protein (1 mg/mL) was emulsified with Freund’s complete adjuvant for the first immunization, emulsified with Freund’s incomplete adjuvant for the subsequent three immunizations. After the last immunization, the titer of the antibody against PCV2-Cap protein in the immunized camel serum was analyzed with an iELISA, using the recombinant PCV2-Cap protein (400 ng/well) as the coating antigen. Preimmune serum was used as a negative control. After incubation with camel serum, rabbit anti-camel IgG antiserum (dilution 1:2000) was added as the secondary antibody, and HRP-conjugated goat anti-rabbit IgG was used as the enzyme-labeled antibody.

One week after the last immunization, 300 mL camel peripheral blood was collected. Peripheral blood mononuclear cells (PBMCs) were isolated with Leucosep® tubes (Greiner Bio-One, Germany). Total RNA was extracted using RNeasy® Plus Mini RNA Extraction kits (QIAGEN Bioinformatics, Germany), and cDNA was synthesized by reverse transcription. VHH fragments were amplified using nested PCR. The first round of PCR amplification was carried out with primers CALL001 and CALL002 (Table S1), and the product of first-round PCR amplification (about 700 bp) was recovered and used as the template for the second round PCR amplification with primers VHH-FOR and VHH-REV [26–28] (Additional file 1: Table S1). Subsequently, recovered VHH fragments (about 400 bp) were inserted into the modified pCANTAB 5E vector[28] with *Pst* I and *Not* I restriction sites, and the recombinant phagemids were transformed into freshly prepared *E. coli* TG1 competent cells by electroporation. The number of transformants was determined by plating cells on LB plates containing 2 % glucose and 100 µg/mL ampicillin and cultured at 37 °C for 8 to 12 hours. On the second day, the colonies were scraped from the plates with a cell scraper, tested with primers p5E-For and VHH-REV(Table S1), and stored at − 80 °C in LB supplemented with 20 % glycerol.

### Panning and identification of PCV2-Cap protein specific nanobodies

**Scheme 1:**
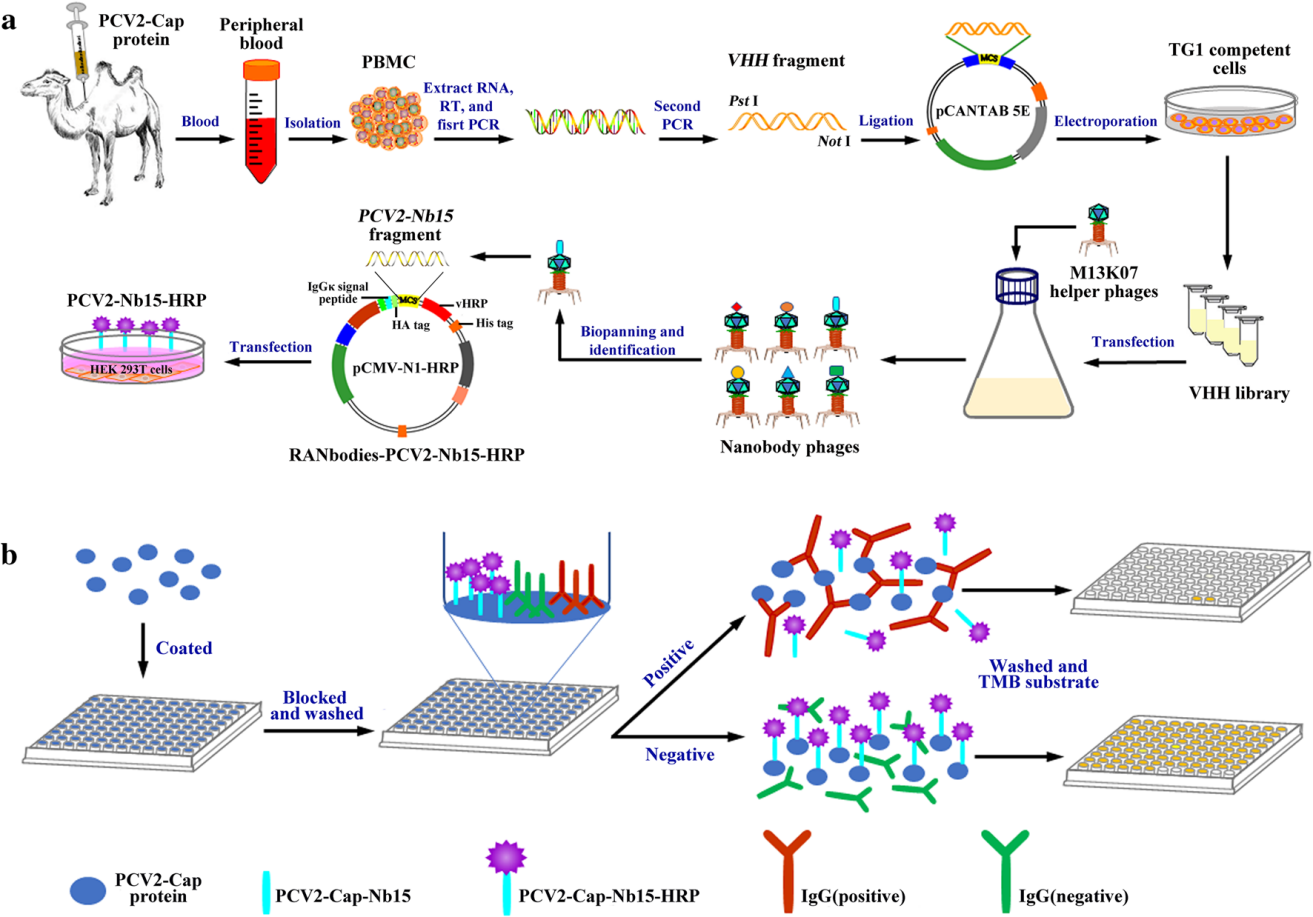
Graphic abstract. **a** Diagram for the acquisition of the VHH library and the expression of PCV2-Nb15-HRP fusion protein. **b** Designation of the developed cELISA

To select specific nanobodies against PCV2-Cap protein, the phage rescue and titration was performed as described previously [29]. Then a 96-well plate (Maxisorp) was coated with PCV2-Cap protein (100 µg/mL) diluted in PBS (100 µL/well) overnight at 4 °C, and PBS was used as a control. On the next day, after washing with PBS containing 2.5 % Tween-20 (PSB’T, V/V) and blocking with PBS’T containing 2.5 % skim milk (SM-PBS’T, W/V), 5 × 10^10^ PFU rescued phage in 100 µL SM-PBS’T was added and incubated for 2 h at RT. After washing 10 times with PBS’T and then 5 times with PBS, 0.1 mol/L freshly prepared triethylamine (100 µL/well) was added to each well and incubated 10 min at RT to elute specific phage particles. Then the eluates were collected and quickly neutralized with equal volume Tris-HCl (1 mol/L, pH 7.4). Fresh *E. coli* TG1 cells were infected with eluted phages and the recombinant phagemids were concentrated with PEG-NaCl for further rounds selection. After three rounds of biopanning, input and output phages were quantified by serial dilution of every round panning and the enriched phage particles were detected using an iELISA with an anti-M13 antibody. Then 96 colonies were randomly picked up for further analysis. Expression of soluble VHHs with an E-Tag in the 96 colonies was separately induced with 1 mmol/L IPTG (TAKARA, Japan). The recombinant VHHs-E-Tag proteins were extracted using an osmotic shock protocol from the periplasm[27] and tested for its capacity binding with PCV2-Cap protein using an iELISA with an anti-E-Tag antibody (HRP) (Abcam-ab3415, UK). Finally, all VHH genes from the positive clones were sequenced, and the nanobodies were grouped according to their CDR3 sequences. The specificity and binding capacity of the nanobodies were detected using the iELISA with an anti-E-Tag antibody[26–28] (Scheme 1a).

### Production of nanobody‑HRP fusion protein against PCV2-Cap protein

The platform for the production of nanobody-HRP fusion proteins was constructed as previously described [26]. PCV2-Cap protein nanobody (Nb15) gene fragment was inserted between the HA tag and HRP sequences of the novel vector pCMV-N1-HRP. The positive recombinant plasmids were confirmed by sequencing and named as RANbodies-PCV2-Nb15-HRP. To produce nanobody-HRP fusion protein, the mammalian cell line HEK 293T cells were transfected with RANbodies-HRP-PCV2-Nb15 plasmid using polyetherimide (PEI, Polysciences Inc. Warrington, USA) reagent. After the cells were transfected for 3 days, the medium containing secreted nanobody-HRP fusion protein was harvested and filtered through 0.45 µm pore cellulose acetate membranes for direct use (Scheme 1a).

To detect the titer of the nanobody-HRP fusion protein in the medium, the filtered medium was directly used for iELISA. Briefly, the wells of the ELISA plate were coated with 400 ng/well purified PCV2-Cap protein overnight at 4 °C, after blocking and washing, a diluted medium was added and incubated. After washing, tetramethylbenzidine (TMB) was added for a colorimetric reaction at RT for 15 min. Finally, the colorimetric reaction was stopped by adding 3 mol/L H_2_SO_4_ (50 µL/well), and the OD_450nm_ values were read using an automated ELISA plate reader (Bio-Rad, USA).

### Establishment of a competitive ELISA (cELISA) using nanobody-HRP fusion protein 

Using the nanobody-HRP fusion protein, a cELISA method for rapid detection of anti-PCV2 antibodies was established. To achieve optimal cELISA performance, various experimental conditions were optimized.

Firstly, the optimal coating concentration of PCV2-Cap protein (50, 100, 200, 300, and 400 ng/well) and the optimal dilution of nanobody-HRP fusion protein (from 1:100 to 1:3200) were determined with direct ELISA using checkerboard titration. The optimal condition was considered when the OD_450nm_ value was close to 1.0.

Secondly, three separate positive and negative porcine sera for anti-PCV2 IgG antibodies were used to optimize dilution of the tested porcine sera. The sera were diluted 1:5, 1:10, 1:20, 1:40, 1:80, and 1:160 for cELISA detection.

Lastly, the competition time for testing serum antibody and nanobody-HRP fusion protein with antigen (15, 30, 45, and 60 min) and the time for colorimetric reaction (10, 15, and 20 min) were optimized. The optimal amount of coated antigen and dilution of nanobody-HRP fusion protein were used. The optimal conditions were determined when the smallest ratio of OD_450nm_ values between the positive and negative serum (P/N) were obtained.

After the conditions were determined, the procedure of the developed cELISA was performed as follows. 96-well plates were coated with the optimal concentration of PCV2-Cap protein and incubated overnight at 4 °C. The plates were then washed 3 times with PBS’T and blocked with SM-PBS’T at 37 °C for 1 h. After washing three times with PBS’T, 100 µL testing mixture containing optimal diluted serum sample and nanobody-HRP fusion protein in SM-PBS’T was added and incubated optimal time at 37 °C. After washing three times with PBS’T again, 100 µL TMB was added to each well and then incubated for the optimal time at RT in the dark. The colorimetric reaction was terminated by adding 50 µL 3 mol/L H_2_SO_4_ to every well. The OD_450nm_ values were then read using an automated ELISA plate reader (Scheme 1b).

The OD_450nm_ value was converted to a percent inhibition (PI) using the following formula: PI (%) = [1 − (OD_450nm_ value of testing serum samples/OD_450nm_ value of negative serum samples)] × 100 %. Then 48 negative serum samples confirmed by a commercial ELISA kit(Shenzhen Finder Biotech Co., Ltd) and the Western blot method were used to determine the cut-off value between the positive and negative serum samples. The cut-off value for the developed cELISA was set at the mean PI of 48 negative serum samples plus 3 standard deviations (SD), which could give either 95 % or 99 % confidence for the negative serum samples fell within the defined range [30].

### Validation of the competitive ELISA

Three hundred and sixty serum samples collected from PCV2 vaccine immunized pigs or non-immunized pigs and confirmed by commercial ELISA kits (Shenzhen Finder Biotech Co., Ltd)were used to assess the sensitivity and specificity of the established cELISA. Besides, 6 positive sera twice diluted from 1:10 to 1:640 were detected with the established cELISA to determine the lowest detection limit. To analyze the cross-reaction of the developed method, a cross-blocking assay was performed by testing the known reference positive sera against porcine reproductive and respiratory syndrome virus (PRRSV), porcine pseudorabies virus (PRV), classical swine fever virus (CSFV), transmissible gastroenteritis virus (TGEV), and porcine epidemic diarrhea virus (PEDV).

The reproducibility of the developed cELISA was evaluated with 6 PCV2-seropositive samples and 3 PCV2-seronegative samples. The coefficient of variation (CV) was used to evaluate the inter-plate differences and the intra-plate differences. Each sample was tested with three different plates on different occasions to determine the inter-assay CV and three replicates within each plate used to calculate the intra-assay CV.

### Comparisons of the cELISA with the commercial ELISA kit

To evaluate the consistency of the cELISA with the commercial ELISA kit, 620 clinical serum samples from immunized pigs of large-scale farms in Shaanxi, Hebei, Shandong, and Henan provinces were tested with the cELISA and the commercial ELISA kits, respectively. The coincidence rate of the results from the two methods was calculated. Sera that the detection results of the two methods did not match were further tested with the Western blot method. In Western blot assay, the purified PCV2-Cap protein was subjected to SDS-PAGE, after the PVDF membrane was imprinted, the different sera (1:500 dilution) were incubated respectively and HRP-conjugated goat anti-porcine IgG (1:5000 dilution) (Jackson ImmunoResearch Laboratories, USA)was then used for detection. After 2 minutes of colorimetric reaction by ECL, the results were displayed using a chemiluminescence imager (Bio-Rad Laboratories, USA).

### Statistical analysis

Statistical analysis and drawing were performed using GraphPad Prism software 8.4 (GraphPad Software, Inc. LA Jolla, CA, USA)using a one-way analysis of variance(one-way ANOVA) followed by Turkey : compare all pairs of columns. Comparisons between groups were considered statistically significant at *p* < 0.05. The Kappa values were calculated to estimate the coincidence between the cELISA and the commercial ELISA kit using SPSS software (Version 20, http://www.spss.com.cn).

## Result

### Preparation of rabbit anti‐camel IgG antiserum

**Fig. 1:**
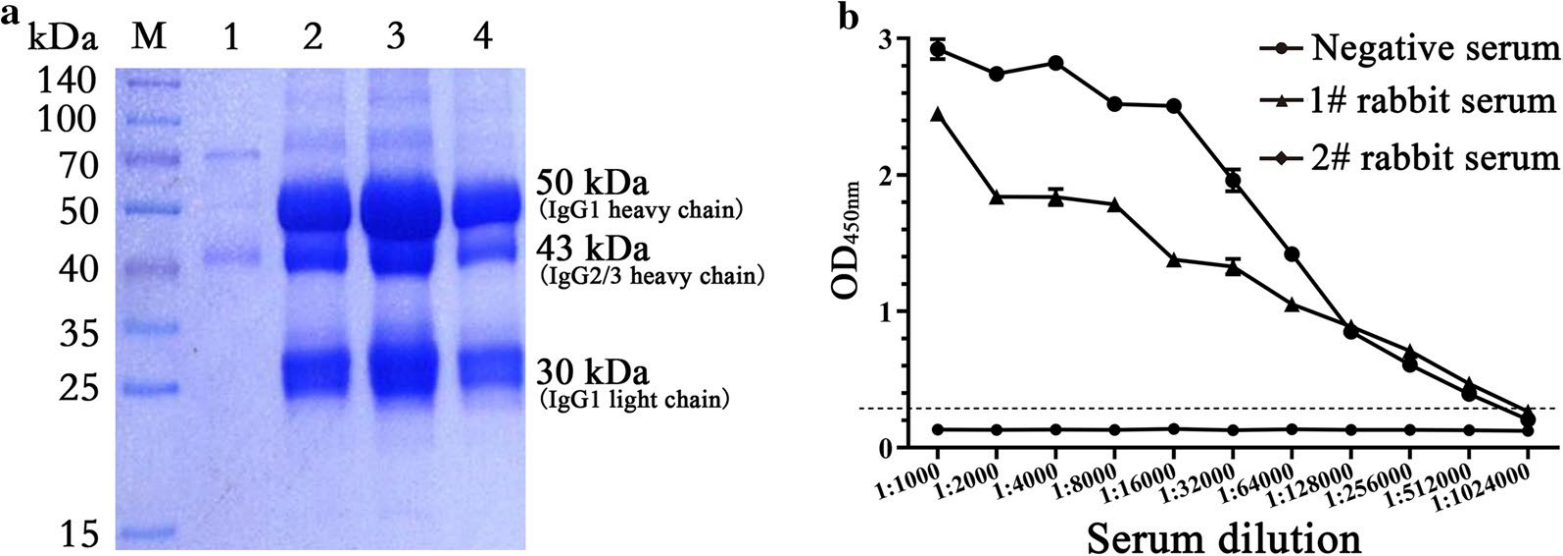
Purification of Camel serum IgG and preparation of its antibody. **a** SDS-PAGE and Coomassie brilliant blue staining analysis of the purified camel serum IgG. M, Prestained protein marker; Lane 1, Wash buffer; Lane 2–4, purified camel serum IgG. **b** Titers of antibodies against camel serum IgG

To detect the antibody titer in immunized camel sera, the rabbit anti-camel IgG was prepared. The SDS-PAGE result showed that the purified camel serum IgG contained two types of IgG, which are the traditional IgG1 about 50 kDa, and the heavy chain antibody IgG2/3 about 43 kDa (Fig. 1a). The result is consistent with previous reports [1]. The rabbits were immunized with purified camel serum IgG, and the rabbit anti-camel IgG antibody titer was detected with iELISA. The result showed that the antibody titer reached 1:512,000 (Fig. 1b).

### A phage display VHH library construction

**Fig. 2:**
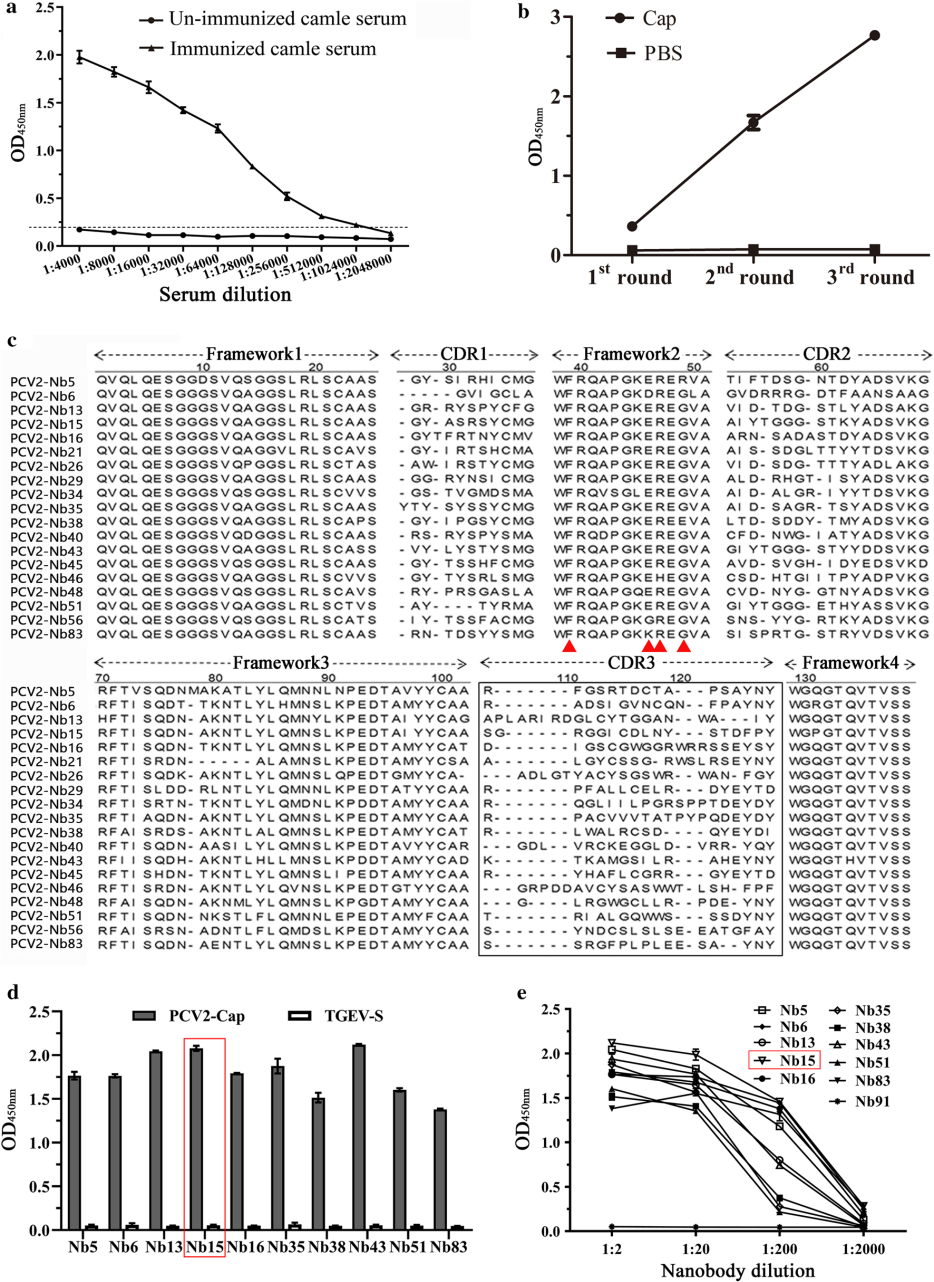
Screening nanobodies against PCV2-Cap protein.** a** Titer of antibody against PCV2-Cap protein in the serum from the immunized camel. **b** Detection of specific phage enrichment with ELISA. **c** Alignment of amino acid sequence of 19 screened nanobodies. The residues at positions 37, 44, 45, and 47 are indicated by red triangles. **d** Specific reactions between the 10 screened nanobodies and PCV2-Cap protein. TGEV-S protein was used as a His-tag control protein. **e** Titration of the 10 screened nanobodies binding with the PCV2-Cap protein. Nb91, a screened negative nanobody was used as negative control

After the last immunization, the titer of antibody against PCV2-Cap protein in camel serum was determined using an iELISA. It reached 1:1,024,000 (Fig. 2a), indicating that the camel produced a good immune response to PCV2-Cap protein. According to previously described methods, total RNA extracted from camel PBMCs was reverse transcribed into cDNA and the VHH genes (approximately 400 bp) were amplified using nested PCR. Cloned VHH gene fragments were inserted into a modified pCANTAB 5E phage display vector. A phage display VHH library against PCV2-Cap protein, with a library capacity of 5 × 10^6^, was successfully constructed. Subsequently, 96 clones were randomly selected and identified with the colony PCR method. The results showed that 98 % (94/96) of the VHH gene fragments were successfully inserted into the phage display vector (Additional file 2: Fig.S1). The positive clones were sequenced and aligned according to their sequences; the results showed that the library has a wide diversity.

### Screening and sequencing nanobodies against PCV2-Cap protein

After 3 rounds of panning, the enrichment of phage particles was evaluated by quantifying the P/N values of every round panning and iELISA detection with an anti-M13 antibody. The results showed that the phage particles carrying specific VHHs against PCV2-Cap protein were strongly enriched (Table 1/Fig. 2b). The nanobody extracts from 96 individual colonies were then expressed and screened for binding to PCV2-Cap protein using iELISA and an anti-E-tag antibody. Sixty-eight soluble VHHs extracts were identified for specific binding to PCV2-Cap protein (Additional file 3: Fig.S2). Those positive clones were sequenced. Sequence analysis based on the amino acid sequences of the CDR3 region revealed that 19 nanobodies were obtained (Fig. 2c). The specificity of 10 nanobodies detected with iELISA showed that all 10 nanobodies could react with PCV2-Cap protein, but not with TGEV-S protein, a His-tag control protein (Fig. 2d). Furthermore, all of the 10 nanobodies showed a high binding activity. However, Nb91, a screened negative nanobody, has no binding ability with PCV2-Cap protein (Fig. 2e). Since the Nb15 showed the strongest binding ability with PCV2-Cap protein, it was selected and expressed for further development of a cELISA.

**Table 1:** Enrichment of nanobodies against PCV2-Cap protein from the phages during three rounds panning

Round of panning	Input phage (PFU/well)	Output phage/P (PFU/well)	PBS output/N (PFU/well)	Recovery rate/(P/Input)	Enrichment(P/N)
1st	5 × 10^10^	3.9 × 10^5^	7.2 × 10^4^	7.8 × 10^− 6^	5.4
2nd	5 × 10^10^	5.5 × 10^5^	7 × 10^4^	1.1 × 10^− 5^	7.9
3rd	5 × 10^10^	3 × 10^6^	2 × 10^3^	6 × 10^− 5^	1.5 × 10^3^

### Expression of PCV2‑Nb15‑HRP fusion protein in HEK 293T cells

**Fig. 3:**
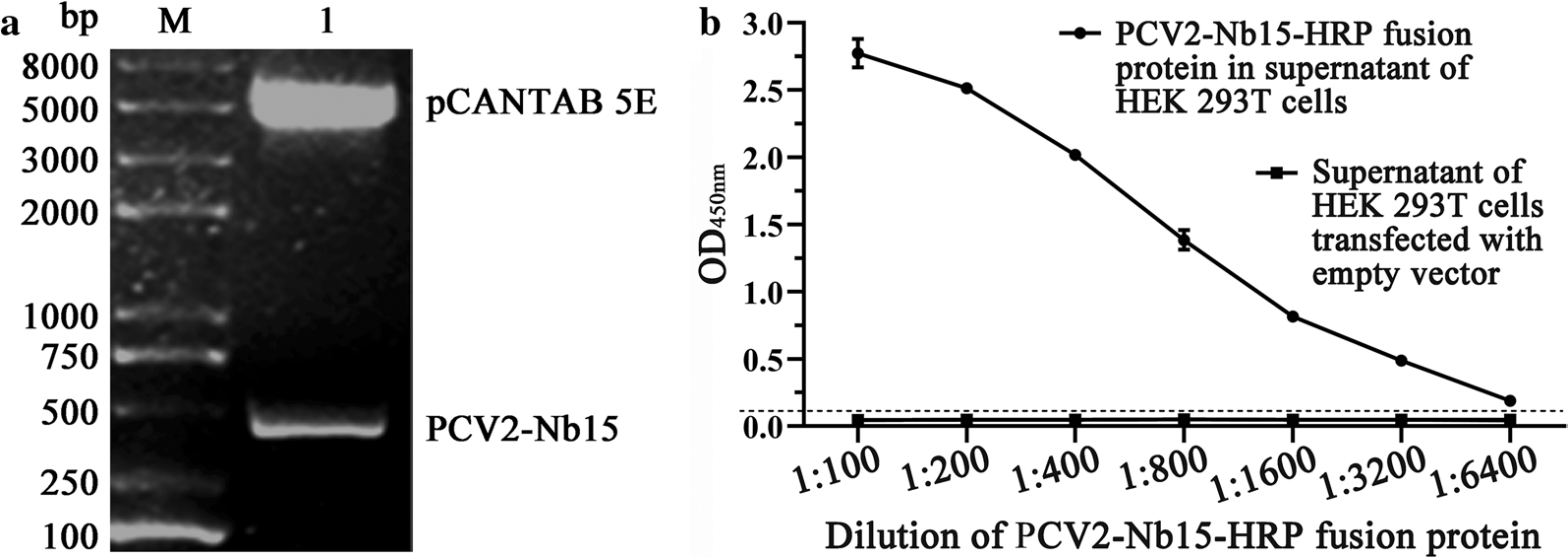
Expression of PCV2‑Nb15‑HRP fusion protein. **a** Obtain PCV2-Nb15 VHH gene by digesting recombinant vector pCANTAB 5E-PCV2-Nb15 with *Not* I and *Pst* I. **b** Detect the reaction of PCV2‑Nb15‑HRP fusion protein with PCV2-Cap protein using indirect ELISA

The PCV2-Nb15 VHH gene was obtained by digesting recombinant vector pCANTAB 5E-PCV2-Nb15 with *Not* I and *Pst* I (Fig. 3a) and inserted into the novel vector pCMV-N1-HRP. After the recombinant plasmid RANbodies-PCV2-Nb15-HRP was transfected into the HEK 293T cells, the amount of PCV2-Nb15-HRP fusion protein in the medium was identified with an iELISA. The result showed that the titer of PCV2-Nb15-HRP fusion protein exceeded 1:6400 (Fig. 3b).

### **Establishment of a cELISA for PCV2 antibody detection using PCV2-Nb15-HRP fusion protein** 

The optimal coating amount of PCV2-Cap protein and the dilution of PCV2-Nb15-HRP fusion protein were determined by checkerboard titration assay. The results showed that the optimal amount of PCV2-Cap protein was 100 ng/well, and the dilution of PCV2-Nb15-HRP fusion protein was 1:800 (Table 2).

**Table 2:** Determination of the optimal coating amount of PCV2-Cap protein and the optimal dilution of PCV2-Nb15-HRP fusion protein

Dilution of PCV2-Nb15-HRP	The OD_450nm_ value of different amounts of PCV2-Cap protein (µg/mL)				
	0.5	1	2	4	8
1:100	2.109 ± 0.062	2.283 ± 0.004	2.536 ± 0.026	2.688 ± 0.049	2.874 ± 0.020
1:200	1.620 ± 0.088	1.962 ± 0.010	2.014 ± 0.034	2.316 ± 0.173	2.804 ± 0.015
1:400	1.170 ± 0.011	1.304 ± 0.018	1.435 ± 0.037	1.684 ± 0.019	2.490 ± 0.014
*1:800*	0.451 ± 0.074	*1.098 ± 0.001*	1.193 ± 0.024	1.35 ± 0.038	1.727 ± 0.029
1:1600	0.264 ± 0.038	0.477 ± 0.009	0.522 ± 0.003	0.686 ± 0.005	1.165 ± 0.053
1:3200	0.180 ± 0.020	0.209 ± 0.008	0.215 ± 0.004	0.396 ± 0.011	0.634 ± 0.011

The italics represent the selected concentration of antigen and the dilution of nanobody

**Fig. 4:**
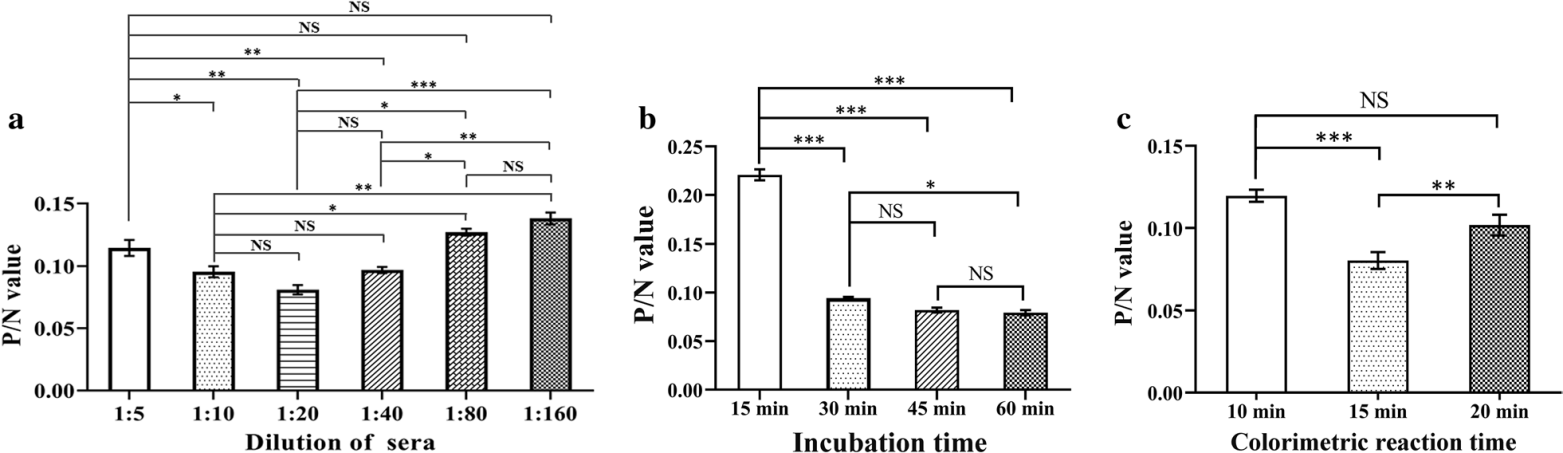
Optimize the reacting conditions of the developed cELISA. **a** Determination of the optimal dilution for serum samples to be tested. **b** Determination of the incubation time of antigen-antibody reaction. **c** Determination of the colorimetric reaction time

The different dilutions of testing porcine sera in the cELISA with positive and negative sera showed that the best dilution of porcine sera was 1:20 (Fig. 4a). Although the P/N value of incubation for 60 min(0.079 ± 0.003) was slightly lower than that of incubation for 45 min(0.082 ± 0.002), there was no significant difference between the P/N value of incubation for 45 min and the P/N value of incubation for 30 min (0.094 ± 0.001)and 60 min(0.079 ± 0.003). After comprehensive consideration, the optimal incubation time for antigen-antibody reaction was determined as 45 min (Fig. 4b). After adding TMB, when the colorimetric reaction time is 15 min, the P/N value is the smallest, and the difference is significant compared with the P/N value of 10 min and 20 min, so the optimal colorimetric reaction time is determined as 15 min (Fig. 4c).

After the conditions were determined, the procedure of the developed cELISA was performed as follows. Firstly, 96-well ELISA plates were coated with 100 ng/well PCV2-Cap protein in 0.01 mol/L PBS (pH 7.2) and incubated overnight at 4 °C. After washing three times with PBS’T, the plates were blocked with 300 µL SM-PBS’T at 37 °C for 1 h. Following three times further washes, 100 µL testing mixtures containing 50 µL serum sample (diluted 1:20 with PBS’T) and 50 µL PCV2-Nb15-HRP fusion protein (diluted 1:400 with PBS’T) were added to the wells and incubated 45 min at 37 °C. After washing, 100 µL TMB was added to each well and the plates were incubated in the dark for 15 min at RT. The colorimetric reaction was stopped by adding 50 µL 3 mol/L H_2_SO_4_ to every well, and the OD_450nm_ values were read using an automated ELISA plate reader.

Then 48 PCV2 antibody negative porcine serum samples were tested using the developed cELISA to determine the cut-off value for the assay. The results showed that the average PI value of the 48 negative serum samples was 7.90 %, with an SD of 4.27 %. So the cut-off value for the developed cELISA was set as 20.72 % (7.90 % + 3SD). If the PI value of the tested porcine serum is equal or greater than 20.72 %, it is considered as positive, and if the PI value is less than 20.72 %, it is considered as negative.

### Specificity, sensitivity, and repeatability of the developed cELISA

**Fig. 5:**
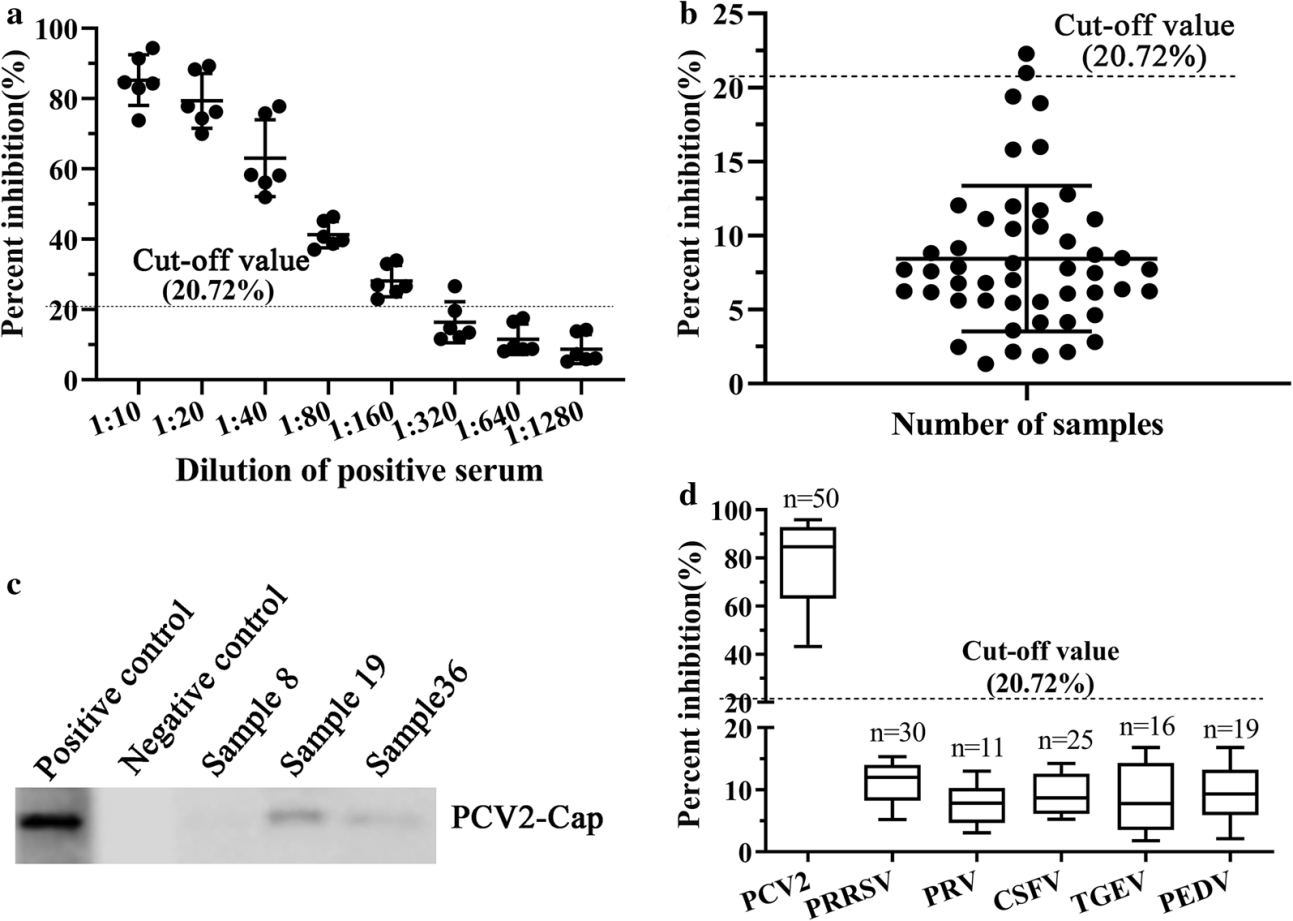
Sensitivity and specificity of the developed cELISA using the PCV2‑Nb15‑HRP as a probe. **a** Determination of the largest dilution for PCV2 antibody-positive porcine sera. **b** Distribution of the PI values when detecting PCV2 antibody-negative porcine sera with the developed cELISA. **c** Identification of the 3 sera with inconsistent detection results using the Western blot method. **d** Evaluation of the developed cELISA by detecting positive sera against other porcine viruses, including PRRSV, PRV, CSFV, TGEV, and PEDV

To determine the sensitivity of the developed cELISA, 311 PCV2 antibody-positive porcine serum samples determined with commercial PCV2 antibody detection kits were measured with the developed cELISA and 310 samples were positive with PI values ranging from 23–96 %. Sample 8 was negative with a PI value of 17.65 %. The PI values of 248 samples were greater than 70 % and only 8 samples had PI values from 23–50 %. So the sensitivity was 99.68 %(310/311) (Table 3). For the different dilution of the 6 positive porcine sera, all samples at the dilution of 1:640 were negative detecting with the developed cELISA, only 1 sample was positive when diluted 1:320 (Fig. 5a). So, for most positive porcine serum samples, the largest dilution was 1:160 for detecting anti-PCV2 antibodies.

**Table 3:** Sensitivity and specificity of the developed cELISA for detecting PCV2 antibody

Samples	Number	Developed ELISA	Commercial ELISA Kit		Sensitivity	Specificity
			+	**−**		
Porcine serum	312	+	310	2	99.68 % (310/311)	95.92 % (47/49)
	48	−	1	47		

To evaluate the specificity of the developed cELISA, 49 PCV2 antibody-negative porcine sera determined with commercial PCV2 antibody detection kits were detected with the developed cELISA. The results showed that 47 samples were negative for anti-PCV2 antibodies with the PI values ranging from 1.33 % to 19.38 % (Fig. 5b). Samples 19 and 36 were positive with PI values of 22.28 % and 20.99 %. So, the specificity of the developed cELISA was 95.92 % (47/49). The 3 samples with inconsistent detection results were detected using the Western blot method. It was found that the results of Western blot were consistent with the cELISA, but not consistent with the results of the commercial kit (Fig. 5c). Furthermore, detection results of positive sera against other porcine viruses were negative with PI values from 1.80 % to 16.81 % (Fig. 5d).

Then, 6 positive sera and 3 negative sera were tested using the developed cELISA. The results of reproducibility showed that the coefficient of variation of PI value in the plate ranged from 1.30–7.40 % with a median value of 4.35 %, and the coefficient of variation of PI value between plates ranged from 3.00–9.00 % with a median value of 6.00 % (Table 4). The coefficient of variation was less than 10.00 %, indicating that the developed cELISA exhibits good reproducibility.

**Table 4:** The results of the repeatability test

Items	Range(%)	Mid-value(%)
Coefficient of variation in the plate	1.30 –7.40	4.35
Coefficient of variation between plates	3.00 –7.23	6.00

### Validation of the developed cELISA

To assess whether the developed cELISA can be used to detect clinical samples, 620 clinical serum samples from immunized pigs of large-scale farms in Shaanxi, Hebei, Shandong, and Henan provinces were detected using the developed cELISA and the commercial PCV2 antibody detection kit, separately. The number of samples with both positive and negative by two methods was 617, so the consistency was 99.52 % (Table 5). Also, statistical analysis showed that the results of cELISA were highly consistent with those of the commercial ELISA kit (Kappa value = 0.907); and the cELISA was more sensitive. Calculation of the PI values of those serum samples and the results showed there were 15 samples with PI values lower than the cut-off value, 17 samples with PI values between 20.71–50 %, 129 samples with PI values between 50–70 %, and 459 samples with PI values greater than 70 %. This indicated that the PCV2 vaccine in the above areas was more comprehensive and the antibody level in pigs was higher after immunization.

**Table 5:** Comparisons of the developed cELISA with commercial ELISA kit by detecting cilinical porcine serum samples

Samples	Number	Developed ELISA	Commercial ELISA Kit		Agreement (%)	Kappa value
			+	**−**		
Clinical porcine serum	605	+	602	3	99.52 % (617/620)	0.907
	15	−	0	15		

## Discussion

PCV2 is considered to be one of the most economically important viral pathogens in pigs. It is a major virulence factor in post-weaning multisystemic wasting syndrome and also causes mixed infections in a variety of diseases[31]. In pigs infected by PCV2, the virus can proliferate within their immune system, causing severe immunosuppression [10, 32]. Therefore, detection, surveillance, and preventive measures for PCV2 infection are important for the swine industry. Immunoperoxidase monolayer(IPMA), immunofluorescence assays (IFAs), and enzyme-linked immunosorbent assay (ELISA) are the most common diagnostic methods for detecting PCV-2 antibodies and the serum neutralization assay is the standard method for the detection of neutralizing antibodies.[22, 33, 34]. But IPMA or IFA assays required an aseptic technique and could take up to 72 h [35]. Since ELISAs may be automated, they are quick and generally sensitive techniques for detecting serum antibodies. Consequently, various PCV2 ELISAs have been described and used in the field for large-scale surveillance. Traditional antibodies, including polyclonal and monoclonal antibodies, have been used to develop ELISA. However, the specificity of polyclonal antibodies is not strong, and the preparation of monoclonal antibodies is complicated, the cost is high, and the enzyme label is difficult. Compared with conventional antibodies, nanobodies have wider antigen-binding sites, higher affinity, and higher specificity [36, 37]. These specificities make them can be used as a probe for the development of ELISA.

In the present study, the nanobodies against PCV2-Cap protein were screened and a PCV2-Nb15-HRP fusion protein was obtained based on the platform constructed in our Lab [26]. Then the fusion protein was used as a probe for developing a cELISA. The establishment of the platform for producing nanobody-HRP fusion protein and the secretory expression of the fusion protein in HEK 293T cells make the fusion protein more active and convenient to produce. Now, we are endeavoring to construct a 293T cell line that stably expresses PCV2-Nb15-HRP fusion protein. Once the cell line has been established, the production of PCV2-Nb15-HRP fusion protein will be easier. The fusion protein used in cELISA saves the use of enzyme-labeled antibodies in traditional methods [38, 39], which greatly saves costs and time. The sensitivity of the developed cELISA was 99.68 % and the specificity was 95.92 %. There was no cross-reaction when detecting positive sera against other porcine viruses. Based on the good sensitivity and specificity observed, the use of the developed cELISA is more advantageous than commercial kits on the market. Furthermore, the PCV2 nanobody can be used with this sensitive and specific method as a high-throughput, promising diagnostic test for the detection of PCV2 antibodies in farmed pigs. Compared with monoclonal antibodies and polyclonal antibodies used in the current ELISAs, the preparation of nanobodies is simpler and more inexpensive. Furthermore, the developed cELISA saves time and can obtain results within an hour.

Besides, the newly developed cELISA was compared with a commercial PCV2 Antibody Test Kit. A total of 620 serum samples collected from immunized pigs of large-scale farms in Shaanxi, Hebei, Shandong, and Henan provinces were tested and the coincidence rate of the two methods was 99.52 % (Table 5), indicating a high correlation between the developed cELISA and the traditional ELISA (Kappa value = 0.907).

## Conclusions

In the present study, we successfully constructed a rich and diverse immune phage display library, and 19 specific nanobodies against PCV2-Cap protein were obtained. Furthermore, based on the obtained nanobodies, PCV2‑Nb15‑HRP fusion protein was expressed in HEK 293T cells and used to develop a novel, rapid, and low-cost cELISA for detecting PCV2 antibody levels in porcine serum samples. This method is time-saving, reproducible, highly sensitive and specific, and has no cross-reactivity with other porcine virus-positive sera. It provides a method for the rapid detection of PCV2 antibody levels and can be widely used.

## Supplementary Information

**Additional file 1: Table S1.** Primer pairs in the study.

**Additional file 2: Figure S1.** Determining VHH genes by colony PCR.

**Additional file 3: Figure S2.** Analysis of the binding ability of recombinant nanobodies against PCV2-Cap protein by indirect ELISA.

## Data Availability

All data generated or analyzed during this study are included in the article and additional file.
