# Correction to: A nanobody-horseradish peroxidase fusion protein-based competitive ELISA for rapid detection of antibodies against porcine circovirus type 2

**DOI:** 10.1186/s12951-021-00815-6

**Published:** 2021-03-03

**Authors:** Yang Mu, Cunyu Jia, Xu Zheng, Haipeng Zhu, Xin Zhang, Haoran Xu, Baoyuan Liu, Qin Zhao, En-Min Zhou

**Affiliations:** 1 Department of Preventive Veterinary Medicine, College of Veterinary Medicine, Northwest A&F University, Yangling, 712100 Shaanxi China; 2 Scientific Observing and Experimental Station of Veterinary Pharmacology and Diagnostic Technology, Ministry of Agriculture, Yangling, 712100 Shaanxi China

## Correction to: J Nanobiotechnol (2021) 19(1):34 https://doi.org/10.1186/s12951-021-00778-8

The original version of this article [1], unfortunately contained some errors.

In the Materials and methods, Subheading ‘Preparation of rabbit anti-camel IgG antiserum’, Paragraph 1, Sentences 7 should read: “Freund’s complete adjuvant (Sigma-Aldrich, **U**SA) was used for the first immunization, followed by Freund’s incomplete adjuvant.”

In the Materials and methods, Subheading ‘Panning and identification of PCV2‑Cap protein specific

Nanobodies’, Paragraph 1, Sentences 3 should read:

“On the next day, after washing with PBS containing **0.05%** Tween-20 (PSB’T, V/V) and blocking with PBS’T containing 2.5% skim milk (SM-PBS’T, W/V), 5×10^10^ PFU rescued phage in 100 µL SM-PBS’T was added and incubated for 2 h at RT.”

The authors apologize for these errors.
