# Supplementary material for: A nanobody‐horseradish peroxidase fusion protein‐based competitive ELISA for rapid detection of antibodies against porcine circovirus type 2
Source: J Nanobiotechnology. 2021 Feb 1;19:34. doi: 10.1186/s12951-021-00778-8 (PMC7852356; doi:10.1186/s12951-021-00778-8)
Supplement: Supplementary file 1 — Additional file 1: Table S1. Primer pairs in the study. [file 12951_2021_778_MOESM1_ESM.doc]

| **Table S1 Primer pairs used in the study** | | |
| --- | --- | --- |
| **Names** | **Sequences (5’-3’)** | **References** |
| CALL001 | GTCCTGGCTGCTCTTCTACAAGG | (Liu et al., 2015; Sheng et al., 2019; Vincke et al., 2012a) |
| CALL002 | GGTACGTGCTGTTGAACTGTTCC |
| VHH-FOR | GATGTGCAGCTGCAGGAGTCTGGRGGAGG |
| VHH-REV | CTAGTGCGGCCGCTGAGGAGACGGTGACCTGGGT |
| p5E-FOR | AATACGCAAACCGCCTCTCC | (Liu et al., 2015) |
