# Supplementary figures and images for: A nanobody‐horseradish peroxidase fusion protein‐based competitive ELISA for rapid detection of antibodies against porcine circovirus type 2
Source: J Nanobiotechnology. 2021 Feb 1;19:34. doi: 10.1186/s12951-021-00778-8 (PMC7852356; doi:10.1186/s12951-021-00778-8)

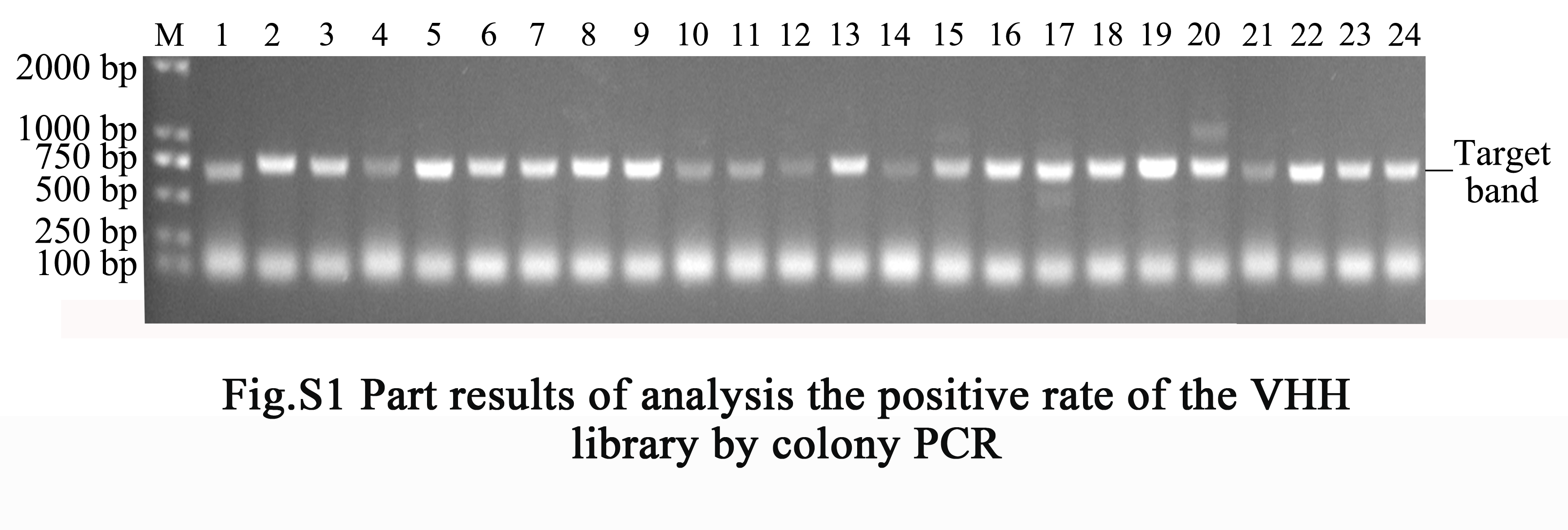

Supplement: Supplementary file 2 — Additional file 2: Figure S1. Determining VHH genes by colony PCR. [file 12951_2021_778_MOESM2_ESM.tif]
